# Synthesis, Structure–Activity Relationships and In Vitro Toxicity Profile of Lactose-Based Fatty Acid Monoesters as Possible Drug Permeability Enhancers

**DOI:** 10.3390/pharmaceutics10030081

**Published:** 2018-07-03

**Authors:** Simone Lucarini, Laura Fagioli, Robert Cavanagh, Wanling Liang, Diego Romano Perinelli, Mario Campana, Snjezana Stolnik, Jenny K. W. Lam, Luca Casettari, Andrea Duranti

**Affiliations:** 1 Department of Biomolecular Sciences, School of Pharmacy, University of Urbino, 61029 Urbino (PU), Italy; simone.lucarini@uniurb.it (S.L.); laura.fagioli@uniurb.it (L.F.); andrea.duranti@uniurb.it (A.D.); 2 Drug Delivery and Tissue Engineering Division, School of Pharmacy, University of Nottingham, Nottingham NG7 2RD, UK; robert.cavanagh@nottingham.ac.uk (R.C.); Snow.Stolnik@nottingham.ac.uk (S.S.); 3 Department of Pharmacology & Pharmacy, The University of Hong Kong, 21 Sassoon Road, Pokfulam, Hong Kong, China; sophiawlliang@gmail.com (W.L.); jkwlam@hku.hk (J.K.W.L.); 4 School of Pharmacy, University of Camerino, 62032 Camerino (MC), Italy; diego.perinelli@unicam.it; 5 Science and Technology Facilities Council (STFC), ISIS Neutron and Muon Source, Rutherford Appleton Laboratory, Didcot OX11 0QX, UK; mario.campana@stfc.ac.uk

**Keywords:** absorption enhancers, sugar-based surfactants, biocompatibility studies, transmucosal drug delivery

## Abstract

Permeability enhancers are receiving increased attention arising from their ability to increase transepithelial permeability and thus, bioavailability of orally or pulmonary administered biopharmaceutics. Here we present the synthesis and the in vitro assaying of a series of lactose-based non-ionic surfactants, highlighting the relationship between their structure and biological effect. Using tensiometric measurements the critical micelle concentrations (CMCs) of the surfactants were determined and demonstrate that increasing hydrophobic chain length reduces surfactant CMC. In vitro testing on Caco-2 intestinal and Calu-3 airway epithelia revealed that cytotoxicity, assessed by 3-(4,5-dimethylthiazol-2-yl)-2,5-diphenyltetrazolium bromide (MTT) and lactate dehydrogenase (LDH) release assays, is presented for most of the surfactants at concentrations greater than their CMCs. Further biological study demonstrates that application of cytotoxic concentrations of the surfactants is associated with depolarizing mitochondrial membrane potential, increasing nuclear membrane permeability and activation of effector caspases. It is, therefore, proposed that when applied at cytotoxic levels, the surfactants are inducing apoptosis in both cell lines tested. Importantly, through the culture of epithelial monolayers on Transwell^®^ supports, the surfactants demonstrate the ability to reversibly modulate transepithelial electrical resistance (TEER), and thus open tight junctions, at non-toxic concentrations, emphasizing their potential application as safe permeability enhancers in vivo.

## 1. Introduction

Sugar-based fatty acid esters usually belong to the class of non-ionic surfactants and possess desirable characteristics suitable for different applications in food, cosmetic and pharmaceutical fields. They are constituted by a sugar moiety as hydrophilic head (polar) linked via an ester bond to a fatty acid chain as hydrophobic tail (non-polar). Various modifications, both on polar head or non-polar tail, have been rationally designed to obtain a broad class of sugar esters with different properties. Particularly, sugar-based fatty acid esters made up of carbohydrate moieties, including mono-, di- or tri-saccharides condensed with saturated or unsaturated fatty acids with different chain lengths have been synthesized to give products with various degree of esterification [1]. Constituted of natural substrates, easily available and therefore inexpensive and renewable, these products are considered ideal raw materials to be employed for a large variety of technological applications such as emulsification, stabilization of disperse systems, solubility or drug permeation enhancement [2,3,4,5].

Sugar-based fatty acid esters are receiving a growing attention due to the large demand for non-toxic, non-irritant and highly biocompatible and biodegradable amphiphilic compounds in different fields [6]. Many studies have recently reported the synthesis and possible applications of selective 6-*O*-sugar fatty acid monoesters, generally referred as sugar monoesters [7,8,9]. Several physico-chemical and biological properties have been studied [10,11], to develop advanced drug delivery systems, including skin penetration enhancing effect or transmucosal permeability enhancement [12,13,14,15]. The use of amphiphilic compounds as permeability enhancers (PEs) for transmucosal drug delivery represents one of the most promising application of surfactants in biopharmaceutics, able to provide a valid alternative (e.g., oral or pulmonary administration) to the conventional (e.g., injection) route of administrations of many therapeutic peptides [16,17,18,19,20].

Among the class of sugar monoesters, sorbitan and sucrose derivatives are those most easily available on the market. However, sugar-based surfactants bearing a different sugar moiety can also represent a valid alternative to the currently available amphiphilic compounds; particularly when industrial waste can be easily transformed in valued-added products. In this respect, the lactose monoester derivatives have been recently brought back into the spotlight, as promising eco-friendly emulsifier and antimicrobial compounds for pharmaceutical, food and cosmetic fields. These surfactants were synthesized for the first time in the 1970s [21], and, over the last decades, they have been proposed, including as excipients in drug formations, additives in the food industry and are being explored for their potential anticancer activity [22]. Despite being well-characterized in term of surface activity [23,24], and emulsifying properties [25], no detailed studies have been performed aimed at defining cytotoxicity profiles of these surfactants. Such information is required to broaden the potential applications of this class of sugar-based amphiphiles for pharmaceutical and cosmetic applications.

The aim of this work was to investigate the relationship between the structure and cytotoxicity of a series of lactose esters derivatives, enzymatically synthesized using saturated fatty acids with different chain lengths (C10; C12; C14; C16). The critical micelle concentration (CMC) was compared with cytotoxicity (IC_50_) evaluated on two selected cell lines as models for the intestinal (Caco-2) and respiratory (Calu-3) epithelia, which are common route of drug administration. In addition, mitochondrial membrane potential, nuclear membrane permeability and effector caspase activation were studied on the same cell lines to deeper understand the mechanism upon cellular toxicity. Finally, transepithelial electrical resistance (TEER) measurements were performed to investigate the effect of saturated acyl chain lactose monoesters on the integrity of the cellular barrier and to provide a preliminary evidence of their transmucosal perturbance action, which is commonly exerted by surfactants acting as permeability enhancers across mucosa.

## 2. Materials and Methods

### 2.1. Materials

Decanoic, lauric, myristic and palmitic acids were purchased from TCI (Zwijndrecht, Belgium), lactose monohydrate from Carlo Erba (Milan, Italy), while Lipozyme^®^ (immobilized from Mucor miehei), *p*-toluenesulfonic acid, 2,2-dimethoxypropane, tetrafluoroboric acid diethyl ether complex, carbonyl cyanide 4-(trifluoromethoxy) phenylhydrazone (FCCP) and all organic solvents used in this study were purchased from Sigma-Aldrich (Milan, Italy). Prior to use, acetonitrile was dried with molecular sieves with an effective pore diameter of 4 Å and toluene was saturated with water. CellEvent™ caspase-3/7 green detection reagent was purchased from Thermo Fisher Scientific (Waltham, MA, USA) and the CellTox™ green cytotoxicity assay acquired from Promega (Madison, WI, USA). The JC-1 probe (5,5′,6,6′-Tetrachloro-1,1′,3,3′-tetraethylbenzimidazolylcarbocyanine, iodide) was purchased from Biotium (Fremont, CA, USA).

### 2.2. Cell Culture Conditions

Caco-2 and Calu-3 cells were obtained from American Type Culture Collection. Caco-2 cells were cultured in advanced Dulbecco Modified Eagle Medium (DMEM) supplemented with 10% *v*/*v* Fetal Bovine Serum (FBS) and 1% *v*/*v* antibiotics-antimycotic. Calu-3 cells were cultured in DMEM F-12 supplemented with 10% *v*/*v* FBS and 1% *v*/*v* antibiotics-antimycotic. All cell lines were maintained at a 5% CO_2_ in a humidified incubator at 37 °C. Caco-2 cells were used between passages 30–45 and Calu-3 cells between 25–40.

### 2.3. Synthesis of Lactose-Based Surfactants

The structures of compounds were unambiguously assessed by ^1^H NMR and ^13^C NMR recorded on a Bruker AC 400 or 100 (Milan, Italy), respectively, spectrometer and analyzed using the TopSpin software package (version 2.1). Chemical shifts were measured using the central peak of the solvent. Column chromatography purifications were performed under “flash” conditions using Merck 230–400 mesh silica gel. TLC was carried out on Merck silica gel 60 F254 plates, which were visualized by exposure to an aqueous solution of ceric ammonium molibdate.

#### 2.3.1. General Procedure for the Synthesis of Lactose Tetra Acetal Monoesters

**Scheme 1:**
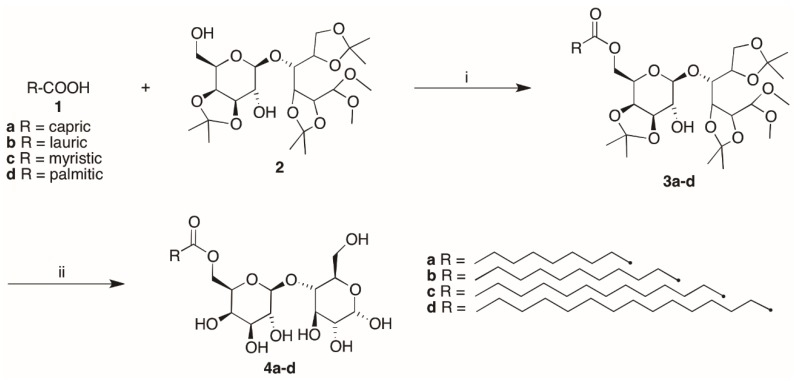
Reagents and conditions: (**a**) toluene, 75 °C, 12 h; (**b**) HBF_4_ Et_2_O, CH_3_CN, 30 °C, 3 h.

Lipozyme^®^ (0.078 g) was added to a solution of the fatty acid (**1a**–**d**) (0.79 mmol) and 4-*O*-(3′,4′-*O*-isopropylidene-β-D-galactopyranosyl)-2,3:5,6-di-*O*-isopropylidene-1,1-di-*O*-methyl-D-glucopyranose (lactose tetra acetate, LTA) [26] (**2**) (0.402 g, 0.79 mmol) in water-saturated toluene at 25 °C [12,27]. The mixture was stirred at 75 °C for 12 h, cooled and diluted with acetone, then the enzyme was filtered, and the filtrate was concentrated. The purification of the residue by column chromatography (cyclohexane/EtOAc 8:2) gave **3a**–**d** as pale yellow oils. (Scheme 1).

*[6′-O-Decanoyl-4-O-(3′,4′-O-isopropylidene-β-d-galactopyranosyl)-2,3:5,6-di-O-isopropylidene-1,1-di-O-methyl-d-glucopyranose]* (**3a**) [27]. Yield: 53% (0.275 g). ^1^H NMR (400 MHz, MeOD) δ: 0.92 (t, 3H, *J* = 6.7 Hz, CH_3_), 1.32 (s, 6H, 2CH_3_), 1.33–1.37 [m, 12H, (CH_2_)_n_], 1.39 (s, 3H, CH_3_), 1.41 (s, 3H, CH_3_), 1.44 (s, 3H, CH_3_), 1.49 (s, 3H, CH_3_), 1.61–1.67 (m, 2H, CH_2_CH_2_COOR), 2.40 (t, 2H, *J* = 7.0 Hz, CH_2_COOR), 3.46 (s, 6H, 2OCH_3_), 3.47 (dd, 1H, *J*_8–9_ = 7.0 Hz, *J*_8–7_ = 8.0 Hz, H^8^), 3.91 (dd, 1H, *J*_4–3_ = 1.2 Hz, *J*_4–5_ = 5.0 Hz, H^4^), 4.04 (ddd, 1H, *J*_11–12__a_ = 1.5 Hz, *J*_11–10_ = 2.1 Hz, *J*_11–12__b_ = 6.8 Hz, H^11^), 4.05 (dd, 1H, *J*_6__b__–5_ = 6.0 Hz, *J*_6__b__–6__a_ = 8.7 Hz, H^6^^b^), 4.08 (dd, 1H, *J*_9–10_ = 5.5 Hz, *J*_9–8_ = 7.0 Hz, H^9^), 4.14 (dd, 1H, *J*_3–4_ = 1.2 Hz, *J*_3–2_ = 7.5 Hz, H^3^), 4.17 (dd, 1H, *J*_6__a–__5_ = 6.0 Hz, *J*_6__a__–6__b_ = 8.7 Hz, H^6^^a^), 4.22 (dd, 1H, *J*_10–11_ = 2.1 Hz, *J*_10–9_ = 5.5 Hz, H^10^), 4.27 (dd, 1H, *J*_12__b–__11_ = 6.8 Hz, *J*_12__b__–12__a_ = 11.5 Hz, H^12^^b^), 4.30 (dd, 1H, *J*_12__a–__11_ = 1.5 Hz, *J*_12__a–__12__b_ = 11.5 Hz, H^12^^a^), 4.31 (ddd, 1H, *J*_5–4_ = 5.0 Hz, *J*_5–6__a_ ≅ *J*_5–6__b_ = 6.0 Hz, H^5^), 4.41 (d, 1H, *J*_1–2_ = 6.2 Hz, H^1^), 4.51 (d, 1H, *J*_7–8_ = 8.0 Hz, H^7^), 4.51 (dd, 1H, *J*_2–1_ = 6.2 Hz, *J*_2–3_ = 7.5 Hz, H^2^) ppm. ^13^C NMR (100 MHz, MeOD) δ: 13.0, 22.3, 24.2, 24.6, 25.1, 25.5, 25.6, 26.2, 27.0, 28.8, 29.00, 29.02, 29.2, 31.6, 33.5, 53.0, 55.1, 63.0, 65.5, 70.8, 73.3, 73.5, 75.4, 76.4, 76.8, 77.6, 79.4, 103.1, 105.7, 108.5, 109.7, 109.9, 173.8 ppm.

*[6′-O-Dodecanoyl-4-O-(3′,4′-O-isopropylidene-β-d-galactopyranosyl)-2,3:5,6-di-O-isopropylidene-1,1-di-O-methyl-d-glucopyranose]* (**3b**) [27]. Yield: 50% (0.274 g). ^1^H NMR (400 MHz, MeOD) δ: 0.92 (t, 3H, *J* = 6.7 Hz, CH_3_), 1.32 (s, 6H, 2CH_3_), 1.33–1.37 [m, 16H, (CH_2_)_n_], 1.39 (s, 3H, CH_3_), 1.41 (s, 3H, CH_3_), 1.44 (s, 3H, CH_3_), 1.49 (s, 3H, CH_3_), 1.62–1.67 (m, 2H, CH_2_CH_2_COOR), 2.40 (t, 2H, *J* = 7.0 Hz, CH_2_COOR), 3.46 (s, 6H, 2OCH_3_), 3.47 (dd, 1H, *J*_8–9_ = 7.1 Hz, *J*_8–7_ = 8.0 Hz, H^8^), 3.91 (dd, 1H, *J*_4–3_ = 1.2 Hz, *J*_4–5_ = 5.0 Hz, H^4^), 4.05 (ddd, 1H, *J*_11–12__a_ = 1.2 Hz, *J*_11–10_ = 2.1 Hz, *J*_11–12__b_ = 6.8 Hz, H^11^), 4.05 (dd, 1H, *J*_6__b–__5_ = 6.0 Hz, *J*_6__b–__6__a_ = 8.7 Hz, H^6^^b^), 4.08 (dd, 1H, *J*_9–10_ = 5.6 Hz, *J*_9–8_ = 7.1 Hz, H^9^), 4.14 (dd, 1H, *J*_3–4_ = 1.2 Hz, *J*_3–2_ = 7.5 Hz, H^3^), 4.17 (dd, 1H, *J*_6__a–__5_ = 6.0 Hz, *J*_6__a–__6__b_ = 8.7 Hz, H^6^^a^), 4.22 (dd, 1H, *J*_10–11_ = 2.1 Hz, *J*_10–9_ = 5.6 Hz, H^10^), 4.27 (dd, 1H, *J*_12__b–__11_ = 6.8 Hz, *J*_12__b–__12__a_ = 11.5 Hz, H^12^^b^), 4.30 (dd, 1H, *J*_12__a–__11_ = 1.2 Hz, *J*_12__a–__12__b_ = 11.5 Hz, H^12^^a^), 4.31 (ddd, 1H, *J*_5–4_ = 5.0 Hz, *J*_5–6__a_ ≅ *J*_5–6__b_ = 6.0 Hz, H^5^), 4.41 (d, 1H, *J*_1–2_ = 6.2 Hz, H^1^), 4.51 (d, 1H, *J*_7–8_ = 8.0 Hz, H^7^), 4.51 (dd, 1H, *J*_2–1_ = 6.2 Hz, *J*_2–3_ = 7.5 Hz, H^2^) ppm. ^13^C NMR (100 MHz, MeOD) δ: 13.0, 22.3, 24.2, 24.6, 25.1, 25.5, 25.7, 26.2, 27.0, 28.8, 29.0, 29.1, 29.2, 29.3, 31.7, 33.5, 53.0, 55.1, 63.1, 65.5, 70.8, 73.3, 73.5, 75.4, 76.4, 76.8, 77.6, 79.4, 103.1, 105.7, 108.5, 109.7, 109.9, 173.8 ppm.

*[6′-O-Tetradecanoyl-4-O-(3′,4′-O-isopropylidene-β-d-galactopyranosyl)-2,3:5,6-di-O-isopropylidene-1,1-di-O-methyl-d-glucopyranose]* (**3c**) [27]. Yield: 44% (0.248 g). ^1^H NMR (400 MHz, MeOD) δ: 0.92 (t, 3H, *J* = 6.7 Hz, CH_3_), 1.30–1.33 [m, 20H, (CH_2_)_n_], 1.35 (s, 6H, 2CH_3_), 1.39 (s, 3H, CH_3_), 1.41 (s, 3H, CH_3_), 1.44 (s, 3H, CH_3_), 1.49 (s, 3H, CH_3_), 1.61–1.67 (m, 2H, CH_2_CH_2_COOR), 2.40 (t, 2H, *J* = 7.0 Hz, CH_2_COOR), 3.46 (s, 6H, 2OCH_3_), 3.47 (dd, 1H, *J*_8–9_ = 7.1 Hz, *J*_8–7_ = 8.0 Hz, H^8^), 3.91 (dd, 1H, *J*_4–3_ = 1.2 Hz, *J*_4–5_ = 5.0 Hz, H^4^), 4.04 (ddd, 1H, *J*_11–12__a_ = 1.0 Hz, *J*_11–10_ = 2.2 Hz, *J*_11–12__b_ = 6.8 Hz, H^11^), 4.05 (dd, 1H, *J*_6__b__–5_ = 6.0 Hz, *J*_6__b–__6__a_ = 8.7 Hz, H^6^^b^), 4.08 (dd, 1H, *J*_9–10_ = 5.6 Hz, *J*_9–8_ = 7.1 Hz, H^9^), 4.15 (dd, 1H, *J*_3–4_ = 1.2 Hz, *J*_3–2_ = 7.5 Hz, H^3^), 4.17 (dd, 1H, *J*_6__a__–5_ = 6.0 Hz, *J*_6__a__–6__b_ = 8.7 Hz, H^6^^a^), 4.22 (dd, 1H, *J*_10–11_ = 2.2 Hz, *J*_10–9_ = 5.6 Hz, H^10^), 4.27 (dd, 1H, *J*_12__b–__11_ = 6.8 Hz, *J*_12__b–__12__a_ = 11.5 Hz, H^12^^b^), 4.30 (dd, 1H, *J*_12__a–__11_ = 1.0 Hz, *J*_12__a–__12__b_ = 11.5 Hz, H^12^^a^), 4.31 (ddd, *J*_5–4_ = 5.0 Hz, *J*_5–6__a_ ≅ *J*_5–6__b_ = 6.0 Hz, H^5^), 4.41 (d, 1H, *J*_1–2_ = 6.2 Hz, H^1^), 4.51 (d, 1H, *J*_7–8_ = 8.0 Hz, H^7^), 4.51 (dd, 1H, *J*_2–1_ = 6.2 Hz, *J*_2–3_ = 7.5 Hz, H^2^) ppm. ^13^C NMR (100 MHz, MeOD) δ: 13.0, 22.3, 24.2, 24.6, 25.1, 25.5, 25.6, 26.2, 27.0, 28.8, 29.0, 29.1, 29.2, 29.31, 29.34, 29.4, 31.7, 33.5, 53.0, 55.1, 63.1, 65.5, 70.8, 73.3, 73.6, 75.4, 76.4, 76.8, 77.6, 79.4, 103.1, 105.7, 108.5, 109.7, 109.9, 173.8 ppm.

*[6′-O-Esadecanoyl-4-O-(3′,4′-O-isopropylidene-β-d-galactopyranosyl)-2,3:5,6-di-O-isopropylidene-1,1-di-O-methyl-d-glucopyranose]* (**3d**) [27]. Yield: 34% (0.200 g). ^1^H NMR (400 MHz, MeOD) δ: 0.92 (t, 3H, *J* = 6.7 Hz, CH_3_), 1.30–1.33 [m, 24H, (CH_2_)_n_], 1.35 (s, 6H, 2CH_3_), 1.39 (s, 3H, CH_3_), 1.41 (s, 3H, CH_3_), 1.44 (s, 3H, CH_3_), 1.49 (s, 3H, CH_3_), 1.60–1.67 (m, 2H, CH_2_CH_2_COOR), 2.40 (t, 2H, *J* = 7.4 Hz, CH_2_COOR), 3.46 (s, 6H, 2OCH_3_), 3.47 (dd, 1H, *J*_8–9_ = 7.1 Hz, *J*_8–7_ = 8.0 Hz, H^8^), 3.91 (dd, 1H, *J*_4–3_ = 1.2 Hz, *J*_4–5_ = 5.0 Hz, H^4^), 4.04 (ddd, 1H, *J*_11–12__a_ = 1.5 Hz, *J*_11–10_ = 2.2 Hz, *J*_11–12__b_ = 6.8 Hz, H^11^), 4.05 (dd, 1H, *J*_6__b–__5_ = 6.0 Hz, *J*_6__b–__6__a_ = 8.7 Hz, H^6^^b^), 4.08 (dd, 1H, *J*_9–10_ = 5.5 Hz, *J*_9–8_ = 7.1 Hz, H^9^), 4.14 (dd, 1H, *J*_3–4_ = 1.2 Hz, *J*_3–2_ = 7.5 Hz, H^3^), 4.17 (dd, 1H, *J*_6__a–__5_ = 6.0 Hz, *J*_6__a–__6__b_ = 8.7 Hz, H^6^^a^), 4.22 (dd, 1H, *J*_10–11_ = 2.2 Hz, *J*_10–9_ = 5.5 Hz, H^10^), 4.27 (dd, 1H, *J*_12__b–__11_ = 6.8 Hz, *J*_12__b–__12__a_ = 11.5 Hz, H^12^^b^), 4.30 (dd, 1H, *J*_12__a–__11_ = 1.5 Hz, *J*_12__a–__12__b_ = 11.5 Hz, H^12^^a^), 4.31 (ddd, 1H, *J*_5–4_ = 5.0 Hz, *J*_5–6__a_ ≅ *J*_5–6__b_ = 6.0 Hz, H^5^), 4.41 (d, 1H, *J*_1–2_ = 6.2 Hz, H^1^), 4.51 (d, 1H, *J*_7–8_ = 8.0 Hz, H^7^), 4.51 (dd, 1H, *J*_2–1_ = 6.2 Hz, *J*_2–3_ = 7.5 Hz, H^2^) ppm. ^13^C NMR (100 MHz, MeOD) δ: 13.0, 22.3, 24.2, 24.6, 25.1, 25.7, 26.2, 27.0, 28.8, 29.1, 29.2, 29.4, 31.7, 33.5, 53.0, 55.1, 63.1, 65.5, 70.8, 73.3, 73.6, 75.4, 76.4, 76.9, 77.6, 79.4, 103.1, 105.7, 108.5, 109.7, 109.9, 173.8 ppm.

#### 2.3.2. General Procedure for the Synthesis of Lactose Fatty Acid Monoesters

Compounds **3a**–**d** (0.25 mmol) were dissolved in tetrafluoroboric acid diethyl ether complex/water/acetonitrile (2.1 mL, 1:5:500) and the mixture was stirred at 30 °C for 3 h [12,27]. The white solids precipitated were then filtered, washed with acetonitrile and dried. The purification by recrystallization from methanol gave **4a**–**d** as white solids. (Scheme 1).

*[6′-O-Decanoyl-4-O-(β-d-galactopyranosyl)-d-glucopyranose, lactose caprate]* (**4a**) [27]. Yield: 75% (0.093 g). ^1^H NMR (400 MHz, DMSO) δ: 0.86 (t, 3H, *J* = 6.6 Hz, CH_3_), 1.20–1.32 [m, 12H, (CH_2_)_n_], 1.48–1.57 (m, 2H, CH_2_CH_2_COOR), 2.31 (t, 2H, *J* = 7.3 Hz, CH_2_COOR), 3.17 (ddd, 1H, *J*_2–1_ = 4.0 Hz, *J*_2–OH2_ = 7.0 Hz, *J*_2–3_ = 9.5 Hz, H^2^), 3.27 (dd, 1H, *J*_4–3_ ≅ *J*_4–5_ = 9.5 Hz, H^4^), 3.33–3.37 (m, 2H, H^8^, H^9^), 3.57 (dd, 1H, *J*_3–2_ ≅ *J*_3–4_ = 9.5 Hz, H^3^), 3.60–3.67 (m, 3H, H^6a^, H^6b^, H^10^), 3.68–3.76 (m, 2H, H^5^, H^11^), 4.09 (dd, 1H, *J*_12b__–__11_ = 4.5 Hz, *J*_12b__–__12a_ = 11.5 Hz, H^12b^), 4.17 (dd, 1H, *J*_12a__–__11_ = 8.5 Hz, *J*_12a__–__12b_ = 11.5 Hz, H^12a^), 4.20–4.25 (m, 2H, H^7^, OH^3^), 4.43 (dd, 1H, *J*_OH6–6a_ ≅ *J*_OH6–6b_ = 6.0 Hz, OH^6^), 4.55 (d, 1H, *J*_OH2–2_ = 7.0 Hz, OH^2^), 4.78 (d, 1H, *J*_OH10–10_ = 5.0 Hz, OH^10^), 4.86 (d, 1H, *J* = 3.0 Hz, OH), 4.90 (dd, 1H, *J*_1–OH1_ ≅ *J*_1–2_ = 4.0 Hz, H^1^), 5.15 (d, 1H, *J* = 3.0 Hz, OH), 6.33 (d, 1H, *J*_OH1–1_ = 4.0 Hz, OH^1^) ppm. ^13^C NMR (100 MHz, DMSO) δ: 14.4, 22.6, 24.8, 28.9, 29.1, 29.2, 29.3, 31.7, 33.8, 60.9, 63.8, 68.7, 70.2, 70.8, 71.7, 72.7, 72.9, 73.3, 81.6, 92.5, 104.0, 173.4 ppm.

*[6′-O-Dodecanoyl-4-O-(β-d-galactopyranosyl)-d-glucopyranose, lactose laurate]* (**4b**) [27]. Yield: 44% (0.058 g). ^1^H NMR (400 MHz, DMSO) δ: 0.86 (t, 3H, *J* = 6.6 Hz, CH_3_), 1.19–1.30 [m, 16H, (CH_2_)_n_], 1.48–1.57 (m, 2H, CH_2_CH_2_COOR), 2.31 (t, 2H, *J* = 7.3 Hz, CH_2_COOR), 3.17 (ddd, 1H, *J*_2–1_ = 4.0 Hz, *J*_2–OH2_ = 7.0 Hz, *J*_2–3_ = 9.5 Hz, H^2^), 3.27 (dd, 1H, *J*_4–3_ ≅ *J*_4–5_ = 9.5 Hz, H^4^), 3.33–3.38 (m, 2H, H^8^, H^9^), 3.56 (dd, 1H, *J*_3–2_ ≅ *J*_3–4_ = 9.5 Hz, H^3^), 3.60–3.67 (m, 3H, H^6a^, H^6b^, H^10^), 3.68–3.76 (m, 2H, H^5^, H^11^), 4.09 (dd, 1H, *J*_12b__–__11_ = 4.5 Hz, *J*_12b__–__12a_ = 11.5 Hz, H^6a^), 4.17 (dd, 1H, *J*_12a__–__11_ = 8.5 Hz, *J*_12a__–__12b_ = 11.5 Hz, H^6b^), 4.20–4.24 (m, 2H, H^7^, OH^3^), 4.43 (dd, 1H, *J*_OH6–6a_ ≅ *J*_OH6–6b_ = 6.0 Hz, OH^6^), 4.56 (d, 1H, *J*_OH2–2_ = 7.0 Hz, OH^2^), 4.79 (d, 1H, *J*_OH10–10_ = 5.0 Hz, OH^10^), 4.86 (d, 1H, *J* = 5.0 Hz, OH), 4.90 (dd, 1H, *J*_1–OH1_ ≅ *J*_1–2_ = 4.0 Hz, H^1^), 5.16 (d, 1H, *J* = 4.0 Hz, OH), 6.34 (d, 1H, *J*_OH1–1_ = 4.0 Hz, OH^1^) ppm. ^13^C NMR (100 MHz, DMSO) δ: 14.4, 22.6, 24.8, 28.9, 29.2, 29.4, 29.46, 29.48, 31.8, 33.8, 60.9, 63.8, 68.7, 70.2, 70.7, 71.7, 72.7, 72.9, 73.3, 81.6, 92.5, 104.0, 173.4 ppm.

*[6′-O-Tetradecanoyl-4-O-(β-d-galactopyranosyl)-d-glucopyranose, lactose myristate]* (**4c**) [27]. Yield: 65% (0.090 g). ^1^H NMR (400 MHz, DMSO) δ: 0.86 (t, 3H, *J* = 6.6 Hz, CH_3_), 1.17–1.32 [m, 20H, (CH_2_)_n_], 1.48–1.57 (m, 2H, CH_2_CH_2_COOR), 2.30 (t, 2H, *J* = 7.4 Hz, CH_2_COOR), 3.16 (ddd, 1H, *J*_2–1_ = 4.0 Hz, *J*_2–OH2_ = 7.0 Hz, *J*_2–3_ = 9.5 Hz, H^2^), 3.27 (dd, 1H, *J*_4–3_ ≅ *J*_4–5_ = 9.5 Hz, H^4^), 3.31–3.37 (m, 2H, H^8^, H^9^), 3.56 (dd, 1H, *J*_3–2_ ≅ *J*_3–4_ = 9.5 Hz, H^3^), 3.60–3.66 (m, 3H, H^6a^, H^6b^, H^10^), 3.67–3.76 (m, 2H, H^5^, H^11^), 4.08 (dd, 1H, *J*_12b__–__11_ = 4.5 Hz, *J*_12b__–__12a_ = 11.5 Hz, H^12b^), 4.16 (dd, 1H, *J*_12a__–__11_ = 8.5 Hz, *J*_12a__–__12b_ = 11.5 Hz, H^12a^), 4.20–4.25 (m, 2H, H^7^, OH^3^), 4.47 (dd, 1H, *J*_OH6–6a_ ≅ *J*_OH6–6b_ = 6.0 Hz, OH^6^), 4.60 (d, 1H, *J*_OH2–2_ = 7.0 Hz, OH^2^), 4.82 (d, 1H, *J*_OH10–10_ = 5.0 Hz, OH^10^), 4.89 (d, 1H, *J* = 4.0 Hz, OH), 4.90 (dd, 1H, *J*_1–OH1_ ≅ *J*_1–2_ = 4.0 Hz, H^1^), 5.19 (d, 1H, *J* = 4.0 Hz, OH), 6.37 (d, 1H, *J*_OH1–1_ = 4.0 Hz, OH^1^) ppm. ^13^C NMR (100 MHz, DMSO) δ: 14.4, 22.6, 24.8, 29.0, 29.18, 29.19, 29.4, 29.48, 29.51, 29.53, 31.8, 33.8, 60.9, 63.8, 68.7, 70.2, 70.7, 71.7, 72.7, 72.9, 73.3, 81.6, 92.5, 104.0, 173.4 ppm.

*[6′-O-Esadecanoyl-4-O-(β-d-galactopyranosyl)-d-glucopyranose, lactose palmitate]* (**4d**) [27]. Yield: 80% (0.116 g). ^1^H NMR (400 MHz, DMSO) δ: 0.86 (t, 3H, *J* = 6.6 Hz, CH_3_), 1.18–1.32 [m, 24H, (CH_2_)_n_], 1.47–1.58 (m, 2H, CH_2_CH_2_COOR), 2.31 (t, 2H, *J* = 7.4 Hz, CH_2_COOR), 3.18 (ddd, 1H, *J*_2–1_ = 4.0 Hz, *J*_2–OH2_ = 7.0 Hz, *J*_2–3_ = 9.5 Hz, H^2^), 3.27 (dd, 1H, *J*_4–3_ ≅ *J*_4–5_ = 9.5 Hz, H^4^), 3.32–3.38 (m, 2H, H^8^, H^9^), 3.57 (dd, 1H, *J*_3–2_ ≅ *J*_3–4_ = 9.5 Hz, H^3^), 3.61–3.67 (m, 3H, H^6a^, H^6b^, H^10^), 3.68–3.76 (m, 2H, H^5^, H^11^), 4.09 (dd, 1H, *J*_12b–11_ = 4.5 Hz, *J*_12b__–__12a_ = 11.5 Hz, H^12b^), 4.17 (dd, 1H, *J*_12a–11_ = 8.5 Hz, *J*_12a__–__12b_ = 11.5 Hz, H^12a^), 4.20–4.28 (m, 2H, H^7^, OH^3^), 4.39 (dd, 1H, *J*_OH6–6a_ ≅ *J*_OH6–6b_ = 6.0 Hz, OH^6^), 4.51 (d, 1H, *J*_OH2–2_ = 7.0 Hz, OH^2^), 4.75 (d, 1H, *J*_OH10–10_ = 5.0 Hz, OH^10^), 4.82 (br s, 1H, OH), 4.90 (dd, 1H, *J*_1-OH1_ ≅ *J*_1–2_ = 4.0 Hz, H^1^), 5.12 (br s, 1H, OH), 6.31 (d, 1H, *J*_OH1–1_ = 4.0 Hz, OH^1^) ppm. ^13^C NMR (100 MHz, DMSO) δ: 14.4, 22.5, 24.8, 29.0, 29.1, 29.2, 29.4, 29.46, 29.50, 31.7, 33.8, 61.0, 63.7, 68.7, 70.2, 70.8, 71.7, 72.7, 72.9, 73.3, 81.6, 92.5, 104.0, 173.4 ppm.

### 2.4. Surface Tension Measurements

Surface tension of different concentrations of surfactant solutions in water was measured using a platinum cylindrical rod probe with wetted length of 1.6 mm (K100-Krüss force tensiometer, Hamburg, Germany) at room temperature. Approximately 1 mL of each surfactant solution was placed onto a Teflon plate and the surface of the liquid was aspirated to remove any remaining impurities. Then, the rod probe was immersed 2 mm into the liquid. Data are expressed as the mean of three repeated measurements performed at room temperature. The critical micelle concentration and the surface tension at the CMC (γCMC) were calculated through the straight-line interception method, while the Gibbs surface excess (Γ_max_) was calculated from the following equation:

(1) $$\Gamma_{\max}=\frac{1}{2.303\;\times \;n\;\times \;R\;\times \;T}\;\left(\frac{\delta_{\gamma }}{\delta \log C}\right)$$

where T is the absolute temperature, R is the gas constant (8.314 J/mol K), *C* is the surfactant concentration, n = 1 for a non-ionic candidate. $\delta_{\gamma }/\delta \log C$ was calculated from the maximum slope of the plot surface tension vs. surfactant concentration in the linear region before CMC.

The minimum area per surfactant molecule at the air-water interface (*A_min_*) was determined as follow:

(2) $$\;A_{\min}=\;\frac{10^{18}}{N\;\times \;\Gamma_{\max}}$$

where *N* is the Avogadro number.

### 2.5. MTT Cell Viability Assays

Caco-2 and Calu-3 cells were seeded in sterile 96-well culture plates at a density of 3 × 10^4^ cells per well. The cells were incubated to attain at least 80% confluence before the experiment. Stock solutions of surfactants were prepared in phosphate buffered saline (PBS). They were diluted at various concentrations (from 0.0078 to 1 mg/mL) in cell culture medium before added to the cells. Complete culture media were used as control. The cytotoxic effect of each surfactant was evaluated using the MTT cell viability assay. After 24 h of incubation, surfactant solution was discarded and replaced by MTT solution (0.8 mg/mL). The cells were subjected to MTT treatment for 2 h. Formazan crystals formed were then dissolved in absolute isopropanol and incubated with gentle shaking at room temperature for 15 min. Absorbance was measured at 570 nm using a microplate reader (Multiskan™ GO Microplate Spectrophotometer Thermo Scientific, Waltham, MA, USA). Percentage of viable cells was calculated using untreated cells as control with 100% cell viability. The percentage of viable cells was plotted against log concentration of the surfactants. IC_50_ (mg/mL), the concentration of surfactant that caused a 50% reduction in cell viability was calculated by fitting the experimental data with dose–response model (Prism 6, Version 6.0b, GraphPad Software).

### 2.6. Lactate Dehydrogenase (LDH) Release Assay

LDH assay was used to evaluate the membrane disruption effect exhibited by the surfactants. Caco-2 and Calu-3 cells were seeded in sterile 96-well culture plates at a density of 3 × 10^4^ cells per well. Surfactant solutions (at the same concentration as in MTT assay described above) were added to the cells and incubated at 37 °C for 24 h. Triton X-100 at 1% *v*/*v* was used as positive control. LDH release assay was conducted according to the manufacturer’s protocol. The percentage of released LDH was calculated relative to the controls by taking samples treated with Triton X-100 as complete LDH release and untreated cells as nil LDH release.

The concentration of surfactant that caused a 50% release of LDH (IC_50,_ mM), was calculated by fitting the experimental data with dose–response model (Prism 6, Version 6.0b, GraphPad Software).

### 2.7. Mitochondrial Membrane Potential (JC-1 Assay)

Caco-2 and Calu-3 cells were seeded in sterile 96 well plates at a density of 1 × 10^4^ cells per well and cultured for 24 h. As above, surfactant solutions were applied in Hank’s balanced salt solution (HBSS) for 24 h. One millimolar FCCP was employed as the positive, mitochondrial depolarizing control. Following exposure, treatments were removed and cells were washed twice with PBS prior to the addition of 50 µL (5 µg/mL) JC-1 dye diluted in complete DMEM (without antibiotics) per well for 15 min at 37 °C. Dye solution was then removed and wells washed with PBS followed by addition of 50 µL/well PBS prior to measuring fluorescence at 550/600 nm (λ_ex_/λ_em_) for detection of JC-1 J-aggregates and 485/535 nm (λ_ex_/λ_em_) for detection of JC-1 monomers. A ratio between JC-1 aggregates and JC-1 monomer signals was then taken, and data normalized by setting the untreated control as a value of 1.0 and the positive control (1.0 mM FCCP) as a value of 0.0.

### 2.8. Caspase 3/7 Activation (CellEvent™ Assay)

Caco-2 and Calu-3 cells were seeded in sterile 96 well plates at a density of 1 × 10^4^ cells per well and cultured for 24 h. Following 24 h exposure on cells, surfactant solutions were removed, and cells washed twice with PBS followed by the addition of 100 µL 1.0% CellEvent™ Caspase-3/7 detection reagent diluted in HBSS buffer per well for 60 min. Fluorescence was then analyzed at 490/540 nm (λ_ex_/λ_em_), and data presented normalized to the untreated control set as a value of 1.0.

### 2.9. Nuclear Membrane Permeability (CellTox™ Green Cytotoxicity Assay)

Caco-2 and Calu-3 cells were seeded at a density of 1 × 10^4^ cells per well in sterile 96 well plates for 24 h prior to assaying. Cells were exposed to treatments for 24 h, followed by the addition of 100 µL (2×) CellTox^TM^ Green reagent (1:500 dilution of CellTox^TM^ Green Dye in Assay Buffer) per well. The resulting solution was incubated at room temperature for 15 min and fluorescence then measured at 495/519 nm (λ_ex_/λ_em_). CellTox^TM^ green signals were normalized by setting the untreated control as 0% and the positive control (1% Triton X-100) as 100% permeabilization of cell nuclei.

### 2.10. Measurement of Trans-Epithelial Electrical Resistance (TEER)

Caco-2 and Calu-3 cells were seeded at a density of 2 × 10^5^ cells per well on filter inserts (Transwell^®^ Permeable Support 12 mm Insert, Corning Life Sciences, Tewksbury, MA, USA) and cultured to confluence under air-liquid interface conditions. Culture media on the baso-lateral sides of the cells were changed every 24 h. For TEER measurements, culture medium was discarded from the cell layer and replaced with Kreb’s Balanced Saline Solutions (KBSS) on both the apical and baso-lateral sides of the monolayers. Cell layers were allowed equilibrating in KBSS at 37 °C, 5% CO_2_ for 45 min prior to sample application. Baseline TEER was measured before the treatment with surfactants. For each surfactant, concentrations that caused 50% cell viability, and the highest concentration that maintained 100% cell viability (according to the MTT assay) were added to the apical sides of the cell layers and incubated for 2 h. TEER was then measured at 5, 30, 60, 90 and 120 min after surfactant addition. Between TEER measurements, cells were incubated at normal cell culture conditions. After 120 min, surfactant was removed, and culture media were added to both sides of the filter inserts. TEER measurement was conducted again after 24 h to evaluate the recovery of cell monolayers. A volt-ohmmeter (Millcell^®^ ERS-2 Voltohmmeter, Millipore, Burlington, MA, USA) equipped with a pair of electrodes was used for TEER measurement. Baseline TEER measured from cell layers incubated in KBSS was used as control and the change in TEER was presented as a percentage relative to baseline value. Three independent experiments were performed in duplicate.

## 3. Results and Discussion

### 3.1. Surface Tension and CMC Determination

**Figure 1:**
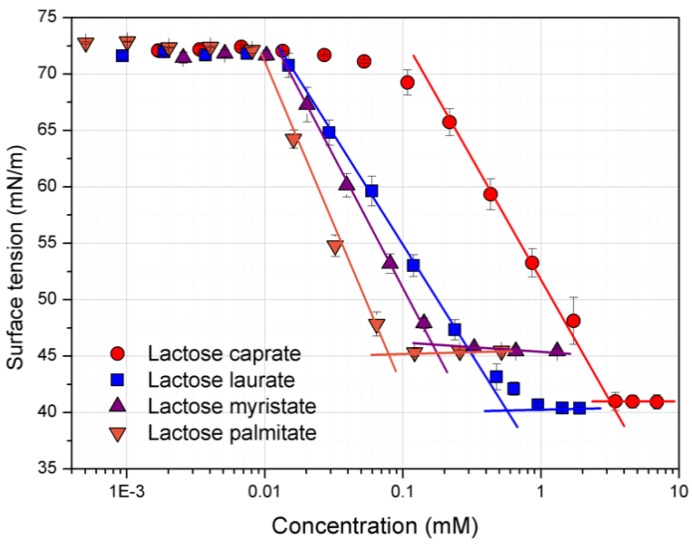
Surface tension (mN/m) vs. concentration (mM) plot for the lactose fatty acid monoester surfactants. Surface tension data are the mean of three repeated measurements (*n* = 3) performed at room temperature. The CMC (critical micelle concentration) is calculated from the straight-line interception method [28].

Figure 1 shows the variation of surface tension over concentrations for each lactose fatty acid monoester. From the plotted curves, it is possible to observe the relevant influence of the carbon chain length on the surface properties of the amphiphiles.

A clear relationship between the calculated CMC values (Table 1) and the carbon chain length can be established.

Indeed, lactose esters with longer carbon chains show a lower CMC because of the higher hydrophobicity. This translates in a lower water solubility and micellization reasonably occurs at lower concentrations. These results agree with those previously reported by Becerra et al. and Garofalakis et al. [24,29].

The surface tension at the CMC (γCMC) was calculated to be around 40 mN/m for lactose caprate and lactose laurate, while it was around 45 mN/m for derivatives with a longer hydrocarbon chain (C14 and C16), demonstrating the potential application of these compounds as surface-active agents (Table 1).

The absorption of the surfactants at the interface is described by the maximum surface excess concentration (Γ_max_), according to the Gibbs isotherm.

The packing ability of surfactants at the interface is influenced by both the hydrocarbon chain length and polar head group. Surfactants with the bulkiest organization are generally characterized by a higher area per surfactants (A_min_). In fact, as previously reported, A_min_ depends not only on the hydrophilic head group dimensions (number of hydroxyl group), but also on packing and stereochemistry of the whole structure [29].

According to Becerra et al., the surface excess is inversely dependent on the hydrocarbon chain length, therefore as the carbon chain length increase, the Γ_max_ decreases. Conversely, the area occupied by each molecule of surfactant increases as the carbon chain length increases [24]. Lactose caprate and lactose laurate, characterized by short hydrocarbon chain lengths, showed the highest Γ_max_, suggesting the organization in a more packed monolayer as indicated by the lower area per molecule.

### 3.2. Biocompatibility Studies

#### 3.2.1. MTT and LDH Assays on Calu-3 and Caco-2 Cells

MTT and LDH assays were carried out to assess the cytotoxicity of various concentration of lactose esters on Calu-3 and Caco-2 cells after 24 h exposure.

**Figure 2:**
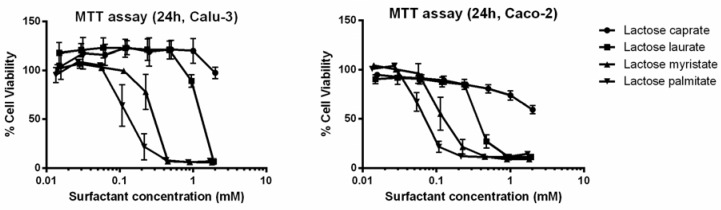
MTT assay of the lactose ester surfactants on Calu-3 and Caco-2 cell lines. The graph plots cell viability (%) as a function of different surfactant concentrations. Data are expressed as mean ± SD (*n* = 3).

Results from the MTT assay (Figure 2) clearly suggest that cell viability of Calu-3 and Caco-2 cells is influenced by both carbon chain length and surfactant concentration. It is interesting to notice how, by increasing the carbon chain length of the synthetized surfactants, cell viability decreases at lower concentrations.

A similar toxicological profile of lactose surfactants was observed when tested on both Caco-2 and Calu-3 cell lines.

**Figure 3:**
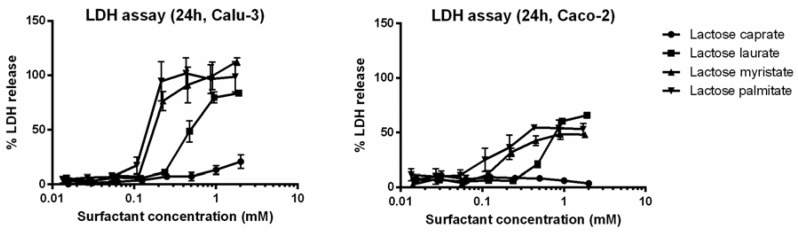
LDH assay of the series of lactose surfactants. The graph plots LDH release (%) from Calu-3 and Caco-2 cell lines as a function of different surfactant concentrations. Data are expressed as mean ± SD (*n* = 3).

Besides the MTT assay, another commonly employed in vitro cytotoxicity assay (LDH assay) was performed. The LDH assay highlights the effect of lactose esters on the cell membrane integrity, confirming the influence of the carbon chain length on the surfactant cytotoxicity on both cell lines (Figure 3).

A comparison on the cytotoxicity of lactose surfactants can be performed by calculation IC_50_ values, which is the concentration of surfactant that causes 50% maximum effect, in terms of 50% reduction of viable cells (MTT assay) or 50% release of LDH (LDH assay) (Table 2).

IC_50_ values decrease from lactose caprate to lactose palmitate independently from the cytotoxicity tests (MTT or LDH assay) or cell lines (Calu-3 and Caco-2). Indeed, for lactose caprate surfactant an IC_50_ value cannot be calculated since it is higher than the tested concentration range, confirming it has the lowest cytotoxicity. For the other lactose surfactants, it is noted that comparable IC_50_ values were calculated from the LDH assay between the two cell lines, while slightly higher IC_50_ values were found in Calu-3 with respect to Caco-2 from the MTT assay.

Interestingly, previous studies demonstrate the relationship between the hydrocarbon chain length and the cytotoxicity of sugar-based surfactants. The cytotoxic effect results from the interplay of different factors such as the ability of the hydrocarbon chain to insert into the lipid bilayer and to perturb membrane as well as the availability of free surfactant molecules, as indicated by CMC value [30,31]. Perinelli et al. recently investigated the correlation between the CMC and the cytotoxicity of *N*-decanoyl amino acid-based surfactants with different polar head groups. The authors demonstrated that toxicity is affected by both the polar head and the hydrocarbon chain length, with the latter parameter causing the main effect [32,33].

Lactose palmitate, which is characterized by the lowest CMC (0.08 mM), demonstrated the highest cytotoxicity in both cell lines. The calculated IC_50_ values for lactose palmitate, were 0.122 mM (Calu-3) and 0.060 mM (Caco-2) for the MTT assay and 0.142 mM (Calu-3) and 0.194 mM (Caco-2) for the LDH assay, respectively.

Moreover, IC_50_ values were compared to the CMC values, since it has already been demonstrated that surfactants with high cytotoxicity, show IC_50_ values lower than the CMC [33,34]. All the surfactants demonstrated IC_50_ values higher or comparable to the CMC in both cell lines (MTT and LDH assays), showing a low toxicity potential of the investigated lactose ester series on the two selected cell lines under defined conditions. This result obtained on cell-based methods in vitro is a preliminary evaluation of the toxicological profile of these surfactants, which should be confirmed by in vivo studies to assess the safety of these amphiphiles for pharmaceutical applications.

#### 3.2.2. JC-1 Assay to Monitor Mitochondrial Health on Caco-2 and Calu-3

To examine the effect of lactose surfactants on the variation of mitochondrial membrane potential (Δ_Ψm_), the ratiometric dye JC-1 assay was employed. The JC-1 dye readily permeates across the cell plasma membrane where it specifically accumulates in active mitochondria, in a potential dependent way. When present in the mitochondria, the dye forms J-aggregates, emitting fluorescence at red 595 nm wavelength, distinct from the dye presents in the cytoplasm which remains in monomeric form emitting fluorescence at green 530 nm wavelength [34]. Mitochondrial depolarization, due to the dissipation of negative charges across the mitochondrial membrane because of mitochondrial disruption, is consequently indicated by a decrease in the J-aggegrate:monomer intensity ratio, thus representing an arbitrary value for Δ_Ψm_.

**Figure 4:**
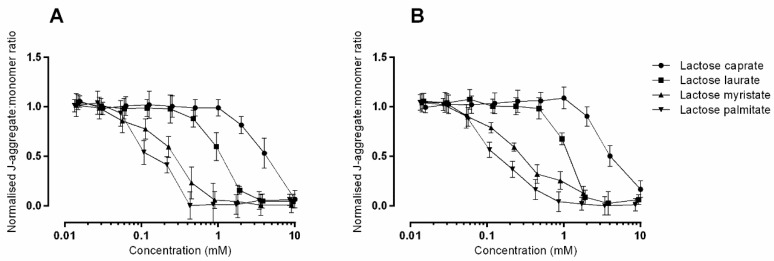
Mitochondrial membrane potential as measured by JC-1 assay in (**A**) Caco-2 and (**B**) Calu-3 cells. Responses are relative to those induced by untreated (HBSS buffer) which were set to a value of 1.0 and to 1.0 mM FCCP (carbonyl cyanide 4-(trifluoromethoxy) phenylhydrazone), known to induce total depolarization of mitochondrial membrane which were set to a value of 0.0. Data represents mean ± SD of triplicates from three independent experiments except for data for Caco-2 dosing of lactose myristate 3.6 mM and 9.0 mM, which comes from one repeat.

The effect of different concentration of lactose surfactants on the mitochondrial membrane potential is reported in Figure 4.

Loss of mitochondrial membrane potential is influenced by both concentration and hydrocarbon chain length of the tested surfactants, thus reflecting the results obtained from the MTT and LDH assays. In general, all the surfactants exert a similar toxicological profile in the two cell lines. When applied at the same concentrations, the highest cytotoxic effect is observed with lactose palmitate, as indicated by the lower J-aggretates:J-monomers ratio.

It is likely that the decline in metabolic activity measured by the MTT assay, observed above (Figure 2), is related to the interruption of mitochondrial respiration that would occur because of mitochondrial membrane potential disruption.

A common cause of mitochondrial depolarization is the induction of apoptosis. Both intrinsic and extrinsic apoptosis cause depolarization, which induces the release of key pro-apoptotic proteins/messengers from the mitochondria.

Depolarization of the mitochondrial membrane potential is commonly caused by the formation of pores or channels across the inner mitochondrial membrane, such as the permeability transition pore that is activated by the pro-apoptotic Bcl-2 family members [35]. Various accounts demonstrate that the dissipation of mitochondrial membrane potential occurs shortly after the permeabilization of the outer mitochondrial membrane [36]. Once the outer membrane is permeabilized, the soluble proteins present in the inner mitochondrial space are released [37,38], many of which play crucial roles in the induction of apoptosis. Therefore, the results gathered suggest the potentially involvement of apoptosis in surfactant toxicity and is explored further in the next section.

#### 3.2.3. Caspase 3/7 Detection

Detection of activated effector caspases 3/7 is a well-recognized marker of programmed cell death. CellEvent™ Caspase-3/7 detection reagent is a four-amino acid peptide (DEVD) conjugated to a specific dye, thus inhibiting its ability to bind to DNA. The conjugated dye is non-fluorescent until the DEVD peptide sequence is recognized and cleaved by activated caspase-3/7, which in turn enables the dye to bind to DNA.

**Figure 5:**
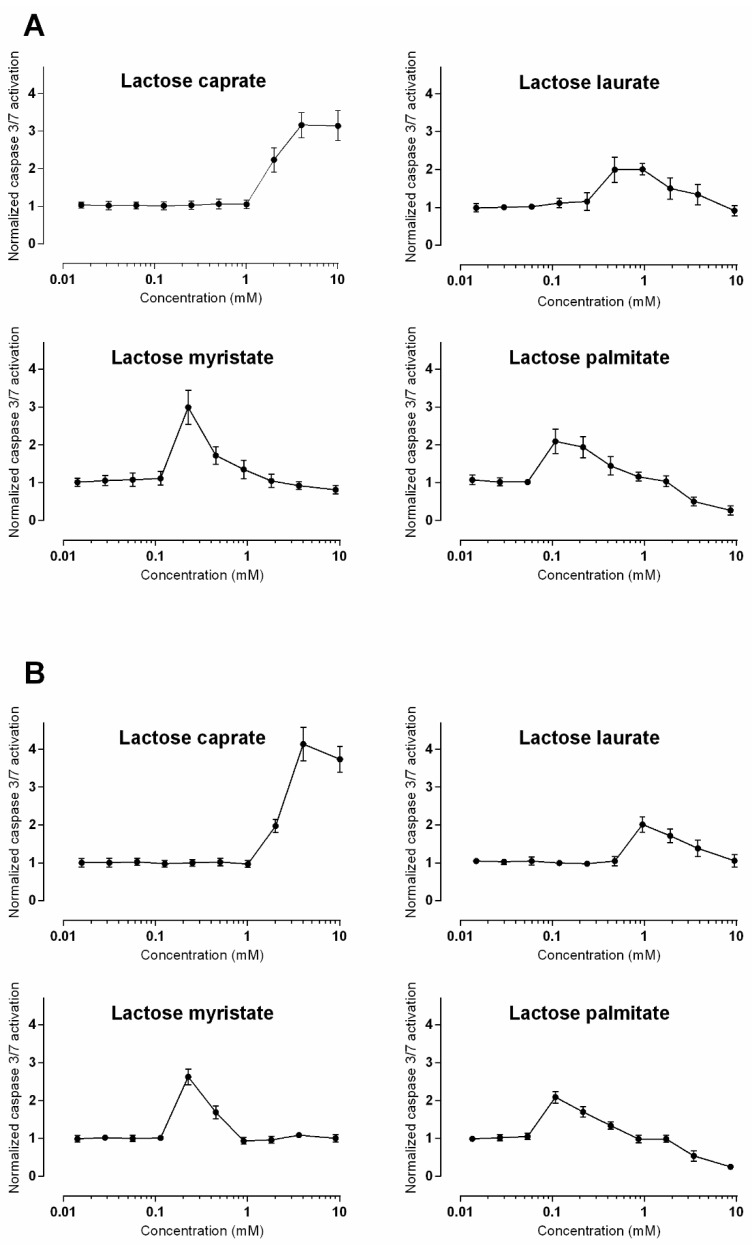
Detection of activated caspase 3/7 in (**A**) Caco-2 and (**B**) Calu-3 cells. Responses are displayed normalized to those induced by vehicle control (HBSS buffer). Data is presented as mean ± SD and represents triplicates from three experiments except for data for dosing of lactose myristate 3.6 mM and 9.0 mM in both Caco-2 and Calu-3 cells, which comes from one repeat.

Figure 5 shows the detection of activated caspase 3/7 induced by the tested lactose surfactants as a function of the applied concentration, in Caco-2 and Calu-3 cells. Based on these results, it is reasonable to assume that the compounds induce caspase 3/7 activation, indicating that apoptosis is occurring. Furthermore, the induction of activated caspases occurs in a compound-dependent manner; therefore, as carbon chain length is increased, the potency to elicit apoptosis increases also since caspase 3/7 activation occurs at lower concentrations. Nevertheless, starting from a certain concentration, the normalized level of caspase 3/7 activation decreases, returning to the baseline values (or lower as is the case for lactose palmitate).

These unusual profiles (Figure 5) observable for all the lactose surfactants may be explained by the dynamic and transient nature of caspase expression. Once cellular death has occurred, indicated by total loss of metabolic activity, the apoptotic process is likely over, therefore cellular expression of effector caspases is no longer necessary. When a cell population undergoes apoptosis, however, caspase levels significantly increase, and the breakdown of the cellular components occurs.

An alternative interpretation to the obtained results that should not be overlooked, is the induction of necrosis at higher concentrations rather than apoptosis, as was observed with quaternary ammonium surfactants [39]. This data set could therefore benefit from the inclusion of an intermediate exposure point (6 or 12 h) which could test the theory based on dynamic caspase expression discussed above. Additional approaches, such as the detection of apoptotic DNA fragmentation, would also aid in deciphering if apoptosis is occurring at higher surfactant concentrations or indeed if necrosis was prevalent.

#### 3.2.4. CellTox^TM^ Green Cytotoxicity Assay

**Figure 6:**
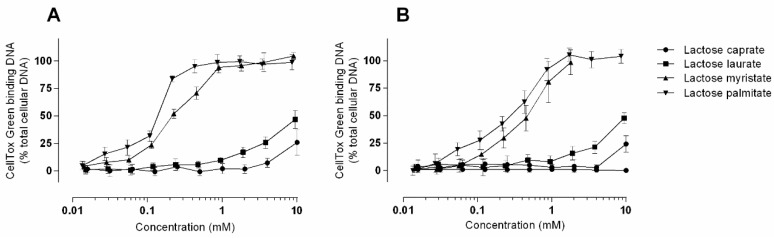
Nuclear membrane permeability as measured using the CellTox^TM^ green assay in (**A**) Caco-2 and (**B**) Calu-3 cells. Responses are relative to those caused by 1.0% Triton X-100, which were set to 100%. Data is presented as mean ± SD and represents triplicates from three independent experiments except for data for Caco-2 dosing of lactose myristate 3.6 mM and 9.0 mM, which comes from one repeat.

CellTox^TM^ green is a DNA binding dye that measures changes in membrane integrity that occurs because of cell death [40]. Signal generation occurs when the cell-impermeable dye binds DNA, and this can occur when DNA is released from cells. Thus, this assay detects for both loss of cellular and nuclear membrane integrity. Alternatively, compounds that enhance membrane permeability can also facilitate dye entry via permeabilization of the cell and nuclear membrane, thus binding to intracellular DNA. Figure 6 shows the results obtained from CellTox^TM^ green assay in Caco-2 and Calu-3 cells treated with different concentration of lactose surfactants. It is possible to observe a concentration and compound dependent increase in nuclear permeability.

Permeabilization of the plasma membrane that may enable the CellTox^TM^ green dye to enter the cell is likely an effect of direct surfactant interaction and incorporation into phospholipid bilayer resulting in altered membrane fluidity, a commonly observed surfactant effect [41]. The increases in nuclear membrane permeability could then subsequently occur as a direct consequence of surfactant incorporation into the nuclear membranes. Additionally, increased permeability in nuclear membranes may be induced because of cellular apoptosis—a late event in apoptosis, mediating the formation of apoptotic bodies. Thus, taken together with the activation of caspase-3/7, increased nuclear membrane permeability at low surfactant concentrations may be occurring in an apoptotic manner. However, at higher concentrations the presence of LDH release alongside increased nuclear membrane permeabilization is likely indicative of surfactant-mediated perturbation of cellular membranes.

### 3.3. Trans-Epithelial Electrical Resistance (TEER) Studies in Calu-3 and Caco-2 Cells

**Figure 7:**
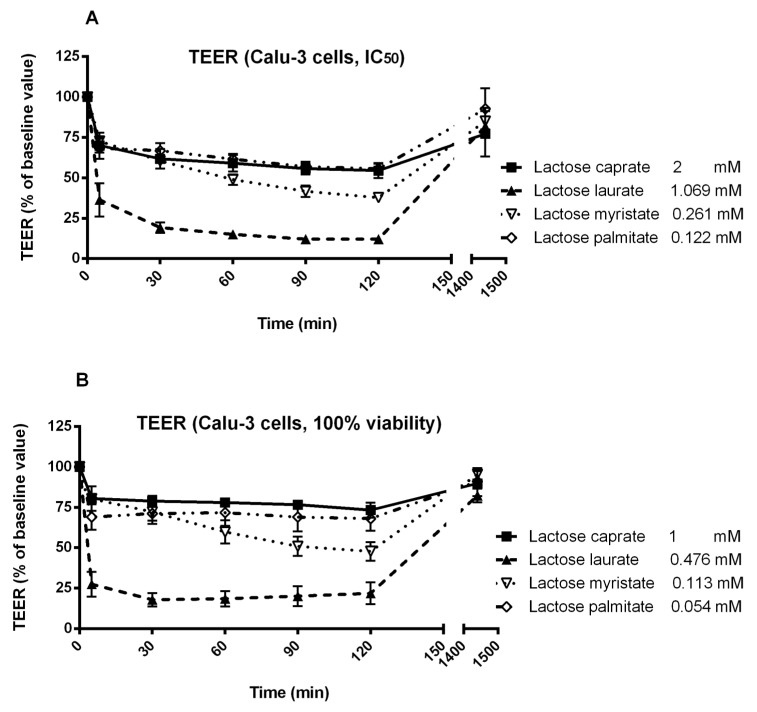
Effect of two different concentrations of lactose surfactants on Calu-3 cell monolayer TEER. (**A**) IC_50_ calculated from MTT assay and (**B**) the highest concentration that shows 100% viability from MTT assay. Data are presented as the mean of three independent experiments.

TEER studies were performed in Calu-3 and Caco-2 cells to evaluate the ability of lactose surfactants to act as absorption enhancers, through transmucosal perturbation possibly by transiently opening tight junctions (Figure 7). Results obtained from TEER measurements should be interpreted carefully with the toxicological profile of the tested compounds to ensure that the changes in TEER was not due to the permeant damage of the membrane integrity. In fact, a transient modulation of tight junction opening commonly translates in a reversible effect on the TEER, while a permanent perturbation of the membrane integrity, on the other hand, is evidenced by a non-reversible effect on the TEER. However, it should also be noted that reversible effect on TEER could be a consequence of mechanisms other than tight junction opening.

In the present study, two concentrations of each lactose surfactant were selected for TEER studies, specifically the IC_50_ calculated from MTT assay and the highest concentration that shows 100% viability in MTT assay, to evaluate the potential application of lactose surfactants within this range of concentrations. Hence, the effect observed on TEER for each surfactant must be correlated with the specific applied concentration, in addition to the structure of the compound (hydrocarbon chain length).

Regarding Calu-3 cells, lactose laurate was found to be the most effective surfactant in decreasing the TEER at both tested concentrations, while all the other surfactants showed only a moderate decrease in the TEER. Lactose laurate was effective in decreasing the TEER at the lowest applied concentration (0.476 mM; 0.025% *w*/*v*), with an effect comparable to the highest applied concentration (1.069 mM; 0.056% *w*/*v*). On the other hand, lactose caprate, applied at a similar concentration (1 mM), only slightly affected the TEER. It is noted that the surfactants were tested at different concentrations according to their toxicity profile. The ability of lactose laurate to lower the TEER significantly at the tested concentrations indicated that this surfactant exhibits a good balance between safety and efficacy.

Interestingly, TEER reversed to the initial value after 25 h with all the tested surfactants and concentrations, suggesting a transient effect on the membrane permeability, possibly by tight junction opening. To elucidate the mechanism and to confirm whether the surfactants could be serve as permeation enhancers, a macromolecule permeability assay could be performed in future study.

**Figure 8:**
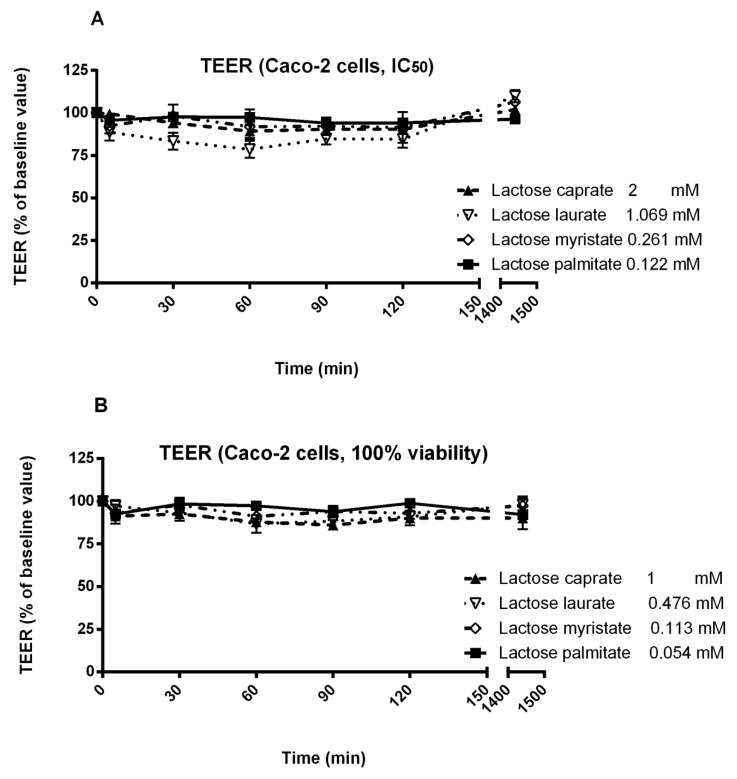
Effect of two different concentrations of lactose surfactants on Caco-2 cell monolayer TEER. (**A**) IC_50_ calculated from MTT assay and (**B**) the highest concentration that shows 100% viability from MTT assay. Data are presented as the mean of three independent experiments.

Regarding Caco-2 cells, different concentrations of surfactants were selected for TEER experiments, based on the IC_50_ calculated from the MTT assay (Figure 8). Due to the higher cytotoxicity displayed on Caco-2 cells, surfactants were tested at lower concentrations.

Caco-2 cells were significantly less sensitive than Calu-3 cells. This was mainly because Caco-2 cells were more prone to the cytotoxic effect of the surfactants, hence a much lower concentrations were used in the TEER study than on Calu-3 cells, rendering the rather poor response on this cell line. Lactose caprate tested at 2 mM, induced a more significant decrease in TEER in Calu-3 cells compared to Caco-2 cells. All the surfactants, tested at their IC_50_ concentration, only poorly affected the TEER, suggesting a reduced efficacy in tight junction opening or other membrane perturbation mechanisms. Nevertheless, lactose laurate showed a more significant effect, with a moderate reduction of TEER. On the other hand, all the surfactants tested at the lowest concentration (100% cell viability from MTT assay) did not induced a significant decrease of TEER.

## 4. Conclusions

This work reports an extensive and comprehensive study of the cytotoxicity profile of selected lactose-based surfactants, as function of their structure (different hydrocarbon chain length) and concentration. Cytotoxicity of the tested surfactants was found to be closely related to their hydrocarbon tail for this specific homologues series in cell cultures tested, thus increasing the hydrocarbon chain length also increased the cytotoxicity. Hence, higher IC_50_ values were observed in presence of more hydrophilic compounds (shorter hydrocarbon chain length). Moreover, comparing CMC and IC_50_ values reveals that the CMCs of the surfactants are lower or similar, thus showing a low toxicity potential on the selected cell lines. Furthermore, the compounds tested showed a comparable cytotoxicity in Caco-2 colonic epithelium and Calu-3 airway epithelium according to both MTT and LDH assay. When applied for 24 h the compounds induced, in a dose-dependent manner, depolarization of the mitochondrial membrane potential and permeabilization of plasma and nuclear membranes, as shown by JC-1 assay and CellTox^TM^ green assay, respectively. The activation of caspase-3/7 suggests apoptosis may play a role in surfactant-induced cell death, particularly at lower surfactant concentrations; however, at higher concentrations, surfactant-mediated membrane perturbation may possibly result in necrotic-like cell death. The initiating factor behind induction of apoptosis remains unknown but is likely associated to alterations in membrane fluidity and the subsequent effect on ion gradient homeostasis and intracellular redox environment. Interestingly, lactose laurate showed a good ability to decrease the TEER at a concentration close to IC_50_ value, while all the other surfactants showed only a moderate effect on membrane perturbation across cell monolayer.

Overall, results from cytotoxicity assays suggest a possible “mild behavior” of lactose monoester surfactants in term of toxicological profile, providing a preliminary evidence regarding one of the possible advantages of using these amphiphiles in different fields.

## Figures and Tables

**Table 1:** Air-water surface parameters (critical micelle concentration (CMC), surface tension at CMC (γCMC), maximum surface excess (Γ_max_) and minimum area per surfactant molecule (A_min_)) of the synthetized lactose fatty acid monoesters determined by tensiometry.

Entry	CMC (mM)	γCMC (mN/m)	Γ_max_ × 10^−6^ (mol/m^2^)	A_min_ (Å^2^)
Lactose caprate	2.58 ± 0.32	40.6 ± 0.04	9.52 ± 0.11	17.46 ± 0.25
Lactose laurate	0.55 ± 0.02	40.4 ± 0.02	9.52 ± 0.05	17.45 ± 0.09
Lactose myristate	0.14 ± 0.05	45.6 ± 0.19	8.37 ± 0.46	19.86 ± 0.80
Lactose palmitate	0.08 ± 0.03	45.1 ± 0.33	6.46 ± 0.11	25.69 ± 0.43

**Table 2:** MTT and LDH cytotoxicity studies of the lactose ester surfactants on Calu-3 and Caco-2 cells. IC_50_ is the concentration of surfactant that causes 50% maximum effect, thus 50% reduction of viable cells (MTT assay) or 50% release of LDH (LDH assay).

Entry	MTT Assay IC_50_ (mM)		LDH Assay IC_50_ (mM)	
	Calu-3	Caco-2	Calu-3	Caco-2
Lactose caprate	>2	>2	>2	>2
Lactose laurate	1.069	0.376	0.452	0.597
Lactose myristate	0.261	0.112	0.189	0.194
Lactose palmitate	0.122	0.060	0.142	0.163
